# Molecular evolution of UCP1 and the evolutionary history of mammalian non-shivering thermogenesis

**DOI:** 10.1186/1471-2148-9-4

**Published:** 2009-01-07

**Authors:** David A Hughes, Martin Jastroch, Mark Stoneking, Martin Klingenspor

**Affiliations:** 1 Max-Planck-Institute for Evolutionary Anthropology, Department of Evolutionary Genetics, Leipzig, Germany; 2 Philipps-Universität Marburg, Faculty of Biology, Department of Animal Physiology, Marburg, Germany; 3 Molecular Nutritional Medicine, Technische Universität München, Else Kröner-Fresenius Center, Freising-Weihenstephan, Germany

## Abstract

**Background:**

Uncoupling protein 1 (UCP1) is a mitochondrial anion carrier, expressed in brown adipose tissue (BAT) of Eutherians. UCP1 is responsible for uncoupling mitochondrial proton transport from the production of ATP, thereby dissipating heat; it is essential for non-shivering thermogenesis (NST) in mammalian BAT. UCP1 orthologs have been identified in non-Eutherian mammals, fish and amphibians. Yet, UCP1 has a unique function in Eutherians in that it is necessary in the production of heat (NST). As such, this study aims to determine the evolutionary mode of UCP1 in Eutherians, where there is clear evidence of UCP1-dependent NST in BAT.

**Results:**

Models of adaptive evolution through phylogenetic analysis of amino acid sequences by maximum likelihood were implemented to determine the mode of UCP1 protein evolution in Eutherians. An increase in the rate of amino acid substitutions on the branch leading to Eutherians is observed, but is best explained by relaxed constraints, not positive selection. Further, evidence for branch and site heterogeneity in selection pressures, as well as divergent selection pressures between UCP1 and its paralogs (UCP2-3) is observed.

**Conclusion:**

We propose that the unique thermogenic function of UCP1 in Eutherians may be best explained by neutral processes. Along with other evidence, this suggests that the primary biochemical properties of UCP1 may not differ between Eutherians and non-Eutherians.

## Background

Uncoupling protein 1 (UCP1) is a mitochondrial protein carrier which, until recently, was thought to be found only in endothermic placental (Eutherian) mammals [1,2]. In Eutherians, UCP1 is the only gene known to be exclusively expressed in brown adipose tissue (BAT), accounting for up to 5% of the total mitochondrial protein in BAT [3]; UCP1 (also known as thermogenin) provides Eutherians, particularly small mammals, hibernators and newborns, with a unique mechanism of non-shivering thermogenesis (NST) [4]. UCP1-dependent NST is probably a feature of most Eutherian mammals, as it has been found recently in the rock elephant shrew, a member of the Afrotherian mammalian lineage which separated early during the evolution of the Eutherians [5]. NST is produced by increasing the proton conductance in the inner membrane of brown adipocyte mitochondria. This increased proton conductance uncouples mitochondrial respiration from ATP synthesis and thereby dissipates the proton motive force as heat [6-9]. It is the high oxidative capacity of mitochondria in BAT and the cellular composition of BAT that allows heat dissipation rates at a power of 300 – 400 W/kg [10-12]. It is these properties of BAT, their mitochondria, and the uncoupling activity of UCP1 which provide Eutherians with NST.

UCP1 is a member of the UCP gene family of mitochondrial transport proteins, which contains two closely related paralogs, UCP2 and UCP3. Although UCP2/3 have been intensively investigated, the molecule(s) that these proteins transport and their overall function remain unclear [6,8,13-16]. It is clear that UCP1 in fish and mammals are orthologs, forming a monophyletic clade, and that the tissue specificity of UCP1 differs between fish, where it is expressed in liver, kidneys and brain, and mammals, where it is expressed exclusively in adipose tissue [2]. Although the function of UCP1 in fish is uncertain, it has been shown that UCP1-like uncoupling activity in fish liver mitochondria is mediated by fatty acids and that it is inhibited by purine nucleotides, analogous to its mediation in Eutherians [17]. Interestingly, while UCP1 expression is increased in response to cold in Eutherians, it is down regulated in the liver of fish in response to cold [2].

In this study we investigate how the evolution of UCP1 may have been influenced by the acquisition of BAT-limited expression and the novel role of NST in Eutherians. Previous work has demonstrated an elevated rate of evolution, i.e. substitution rate, on the UCP1 Eutherian lineage [18]. Yet, what remains uncertain is the mode of evolution which acted upon this lineage. Identification of lineage-specific directional selection would indeed be suggestive of the acquisition of a new function by UCP1. In contrast, the lack of support for lineage-specific directional selection would not rule out this possibility, but may be more suggestive of Eutherian and non-Eutherian UCP1 functional similarities and thus an evolution of NST through neutral processes. We implement a series of phylogenetic models by maximum likelihood to estimate selection pressures at non-synonymous or replacement sites and at synonymous sites [19]. Contrasting the rate of evolution at these two sites (dN/dS or ω) provides a means of evaluating the selection pressures which have acted on the protein during the evolution of UCP1. Our goal is to determine the mode of selection occurring on the Eutherian lineage, where novel biochemical function may have evolved in the course of UCP1 becoming a protein obligated to BAT-limited expression and NST.

## Results

**Figure 1:**
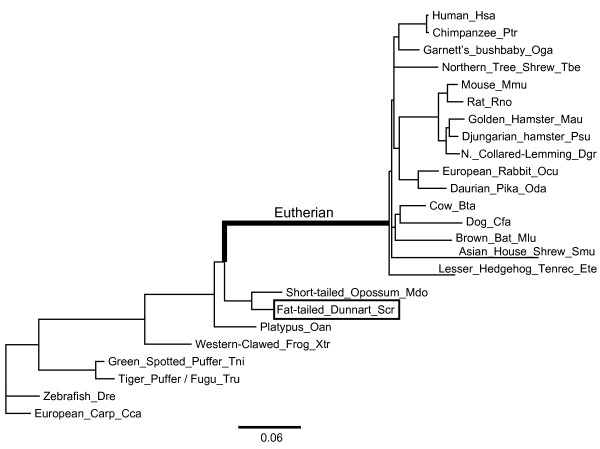
**Branch lengths are estimates of the number of substitutions per codon as performed in the M2 model with the Eutherian branch set as the foreground branch**. Each lineage is defined with the common name of each species and the three letter species code.

Available UCP1, UCP2, and UCP3 sequences were retrieved from 39 species ranging from teleost fishes to mammals (Additional file 1). Nucleotide and protein distance NJ trees, the species tree and a maximum likelihood (ML) tree were generated for the UCP1 locus. No one tree was significantly better than any other as indicated by the RELL method [20] and S-H test [21]. To evaluate the robustness of the results we chose to run all analyses with both the species tree (Figure 1) and the ML tree. As the results were largely concordant between trees and codon substitution models (data not shown), the results using the species tree and the F3X4 codon substitution model are presented in the main text, unless specified otherwise. All analyses for the UCP2 and UCP3 loci used the species tree. Furthermore, all analyses critical for our conclusions were repeated using the alignments from a second alignment software package, namely T-Coffee. Again, this was performed to insure the robustness of our results, as different methods may produce different alignments that then influence the outcome of all subsequent analyses. All results were highly congurent between the two alignment methods; we therefore present parameter estimations derived from alignments using MUSCLE, and present only those derived from T-Coffee which would lead to different conclusions.

**Figure 2:**
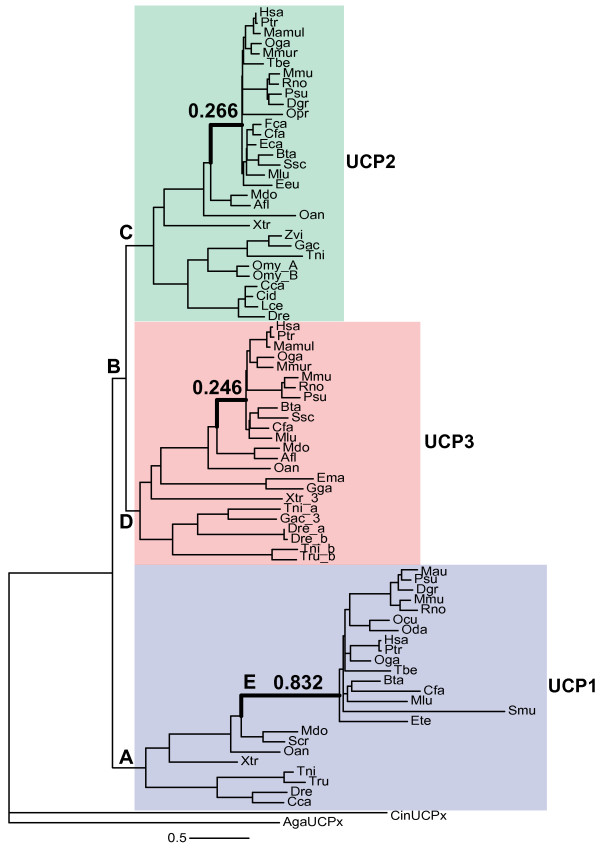
**UCP species tree**. Thick branches are Eutherian lineages for each respective gene. The values above each Eutherian branch are branch length estimates from the M0 model. Three letter species codes are given at the tip of each lineage. Species names can be found in Supplementary Table 1.

The objective here is to determine the mode of selection that has acted on the UCP1 coding sequence in Eutherians, where it is certain that UCP1 is involved in the dissipation of heat essential for non-shivering thermogenesis. We tested for directional selection on the Eutherian branch (Figure 1), and for divergent selection pressure between the Eutherian UCP1 clade and its paralogs UCP2 and UCP3 (Figure 2). This was done by comparing the ω value among lineages and among sites across the UCP1 phylogeny, in order to determine the mode of selection and the magnitude of change in selective constraint (if any) acting across the UCP1 protein as well as within particular functional domains of UCP1.

### Branch and site ω rate variation

The one-ratio model (M0), which constrains ω to be equal across all lineages of the phylogeny, was used to evaluate the global ω value across the UCP1 phylogeny. The UCP1 species phylogeny (Figure 1) was used in this analysis. Global ω was estimated to be 0.124, indicative of strong purifying selection. Omega estimates for UCP2 and UCP3 phylogenies, also using species trees appropriate for the UCP2 and UCP3 data, were 0.072 and 0.083, respectively. Allowing ω to vary among branches across the UCP1 phylogeny (M1 model) results in a significantly improved fit to the data (likelihood ratio test (LRT), 2Δℓ = 217, p < 0.001, df = 44). Thus, there is lineage-specific heterogeneity in ω across the UCP1 phylogeny.

Along with lineage heterogeneity, variation in ω across sites can also occur. Theoretically, different protein domains with different functions may experience different selection pressures, which can be tested by fitting the data to a model comprising different site classes. The M3(discrete) model partitions the sites among three site categories, based on a discrete distribution. Each site category has an estimated proportion (p_0_, p_1_, p_2_) and their ω values (ω_0_, ω_1_, ω_2_) are estimated from the data. This model results in a significantly better fit to the data than the null M0 model (LRT: 2Δℓ = 468, df = 4, p-value < 0.001). This observation is also true for the UCP2 and UCP3 loci (Table 1). The ω estimates vary among sites for each gene; however, in general the fraction of sites with low ω estimates, indicative of strong selective constraints, is reduced for UCP1 relative to UCP2 and UCP3. Only 46% of UCP1 sites are under strong purifying selection (the lowest ω category), compared to 60–61% for UCP2 and UCP3 (Table 1). This is consistent with the previous observation that the M0 ω estimate is higher for UCP1 (0.124) than for UCP2 (0.072) or UCP3 (0.083).

**Table 1:** M3(discrete) sites model for the UCP1, UCP2 andUCP3 phylogenies

	**p_0_**	**ω_0_**	**p_1_**	**ω_1_**	**p_2_**	**ω_2_**	**p-value**
**UCP1**	0.46	0.01	0.43	0.18	0.11	0.48	<0.001
**UCP2**	0.61	0.004	0.31	0.12	0.07	0.65	<0.001
**UCP3**	0.6	0.01	0.3	0.14	0.11	0.5	<0.001

p_0_, p_1_, and p_2 _are the estimated proportion of sites, and ω_0_, ω_1_, and ω_2 _are the estimated ω values, for site class 0 (strong purifying selection), site class 1 (purifying selection), and site class 2 (weak purifying selection), respectively.

### Relaxed constraint on the Eutherian lineage

To determine if the Eutherian UCP1 lineage (Figure 1) has evolved at a different rate, as compared to the rest of the phylogeny, a two ratio model (M2) was implemented. In this model there are two branch categories, and thus two independent estimates of ω: category 0, for the background branches, i.e. all lineages in the phylogeny excluding the Eutherian lineage; and category 1 for the foreground branch, i.e. the Eutherian lineage. In this model the estimated ω_1 _is 0.582 for the Eutherian branch, and 0.113 for the background branches. A LR test indicates that the M2 model provides a significantly better fit to the data that the M0 model (2ΔL = 47.14, p < 0.001, df = 1). Thus, there is a significantly higher ω value for UCP1 on the Eutherian lineage relative to the rest of the phylogeny. However, this estimate is not indicative of positive selection, it is rather indicative of relaxed constraint. By contrast, the UCP2 and UCP3 ω estimates for the Eutherian lineage do not differ from the rest of the phylogeny, as the LR tests do no indicate a better fit for the M2 model over the M0 model for these two genes (UCP2: p = 0.11; UCP3: p = 0.95). This contrast between the Eutherian UCP1 lineage, vs. the Eutherian UCP2 and UCP3 lineages, is further illustrated by the contrast in branch lengths (number of substitutions per codon) in the UCP phylogeny (Figure 2).

In principle, the increased ω estimate on the Eutherian UCP1 lineage could be the result of either an increase in dN or a decrease in dS. To determine the best explanation for this observation we compared the observed dN and dS values for the Eutherian lineage to the distribution of these estimates across the UCP1 phylogeny, given the estimates from the M2 model. The Eutherian UCP1 lineage dS is 0.27, which falls in the 70^th ^percentile of dS values for all lineages in the phylogeny, whereas dN for the Eutherian lineage is 0.16, which is the largest dN value in the UCP1 phylogeny. Hence, it is dN, rather than dS, that appears responsible for the increased ω estimate on the Eutherian UCP1 lineage, again indicative of relaxed constraint.

### Branch-sites model

Branch models have little power to detect directional selection which has acted on just a few codon sites across a lineage, as any signature of selection would be lost in the abundance of negative selection acting across the remaining codons. For this reason, a branch-sites method has been developed that allows variation in ω across individual codons on a specific lineage [22,23]. This model (MA) designates two categories of branches, again foreground and background, where positive selection is modeled only on the foreground branch. Further, the model includes four site categories for both foreground and background (0, 1, 2a, 2b). Class 0 sites are sites under purifying selection, where ω_0 _is estimated to be between 0 and 1. Class 1 sites are neutral sites, where ω_1 _is set to 1. Site classes 2a and 2b are sites which are modeled to be under purifying selection (ω_0_) or neutral evolution (ω_1_), respectively, on the background branches. On the foreground branch, these sites are under positive selection, where ω_2 _is allowed to be greater than 1. We have implemented the MA (branch-sites) model, designating the Eutherian branch of the UCP1 phylogeny (Figure 1) as the foreground branch. The results of this analysis are robust across the different codon substitution models and trees. As done for all analyses, the MA model was tested with both the species tree and the ML tree in combination with the F3X4, F61 and FMutSel models of codon substitution. The MA model (ω_2 _= 1.45, p_2 _= 0.33, ℓ = -10101.60) did not provide a significantly better fit than the null model, where the null model is neutrality (site class 2 is fixed to equal 1, and thus tests if the MA ω_2 _estimate is significantly greater than 1), in any of the analyses (2Δℓ = 0.84, df = 1, p = 0.36). Thus, these analyses do not provide any evidence for directional selection on the Eutherian UCP1 lineage.

Because it is theoretically possible for novel functions or functional divergence to evolve without positive selection [24,25], it may remain informative to functionally investigate the sites identified in these analyses. Each of the branch-sites models do identify a few robust sites which have greater than a 95% posterior probability of being correctly assigned to site class 2, i.e. those which may have experienced selection, where ω is greater than one (but not significantly so as demonstrated in the LRT), by the Bayes empirical Bayes (BEB) method [26]; these sites are: 13M, 19S, 26L, 73K, 121L, 150I, 167T, 221V, 256M. It must be emphasized that this analysis is only considered to be informative when the null model of neutrality is rejected and a model of selection provides a better fit to the data. Thus, there is no support for these sites to have been the subject of selection; yet these sites could prove to be informative for future studies on UCP1 function.

### Reduction of UCP1 evolution in Eutherians

To further investigate the possibility of selection we tested for a relative reduction in dN across the Eutherian UCP1 phylogeny. Following the acquisition of a new function by a protein via positive selection at replacement sites, it is predicted that protein evolution should slow down and be dominated by purifying selection [27,28]. To determine if there was a reduction in the rate of non-synonymous substitutions across the UCP1 phylogeny, after the demonstrated relative increase of dN on the Eutherian lineage, we used the UCP2 and UCP3 phylogenies as comparative loci. In the branch (M2) and branch-sites (MA) analysis neither UCP2 nor UCP3 exhibit evidence for either directional selection or relaxed constraints on the Eutherian branch. The evolutionary history of UCP1 is unique in this respect. Thus we assumed that the estimated evolutionary constraints across the Eutherian phylogenies of the UCP1 paralogs are a good proxy for what may be expected across the UCP1 phylogeny. Estimated dN values, from the M1(free ratio) analysis, for all congruent branches on the UCP1, UCP2 and UCP3 phylogenies were used to perform a one-sided paired Wilcoxon test. If the Eutherian branch had experienced selection at replacement sites we would predict that dN across Eutherians in the UCP1 phylogeny should, at the very least, not be greater than that observed in its paralogs. Contrary to this prediction we find that the rate of substitution at replacement sites remains greater (median dN = 0.01) than that observed across the UCP2 (median dN = 0.003, p-value < 0.001) and UCP3 (median dN = 0.004, p-value = 0.0365) phylogenies. We also found that the rate of replacement substitutions across the UCP3 phylogeny is greater than that estimated in the UCP2 phylogeny (p-value < 0.001). Therefore, congruent lineages across UCP phylogenies do differ from each other in the rate of dN, yet the UCP1 phylogeny has the largest dN rate. There is thus no evidence that UCP1 evolution has slowed in Eutherians, relative to UCP2 or UCP3, as may be expected following a selective event for novel function. Thus these observations refute a conclusion of directional selection and support a model of relaxed constraint on the Eutherian UCP1 lineage.

### Selection for UCP1 on the Therian lineage

Recently, it was discovered that a single marsupial species, the fat-tailed dunnart (*Sminthopsis crassicaudata*), also contains BAT which is recruited in response to cold acclimatization, and that UCP1 expression increases two-fold under cold conditions [1]. Furthermore, the metabolic rate of this species increased in response to norepinephrine treatment [29], consistent with regulation by UCP1. We therefore tested the Therian branch (which includes marsupials but excludes monotremes) for branch-site directional selection on UCP1. We found robust support across all analyses (species tree, ML tree, each codon substitution model and alignments, p < 0.001) for selection acting on a few sites on the Therian lineage. In these analyses, 90% of the sites on the Therian branch are under strong purifying selection with a ω of 0.08, 8.2% of sites are neutrally evolving, and 1.5% of sites have an estimated ω of 8 (i.e., dS was estimated to be equal to 0.0). Two sites, 79P and 178T, were identified with >75% probability by the BEB method of having been the target of directional selection. While it currently remains unknown if UCP1 in the fat-tailed dunnart truly has a thermogenic function, these two identified sites may prove informative in further studies of UCP1 function.

### Functional divergence of UCP1

Directional selection is not a necessary requirement for functional divergence to occur in the evolution of gene families, as a new function for a gene can arise through neutral processes via genetic co-option [30]. To address this possibility of divergent evolution of UCP1 and its paralogs via neutral processes, we used the MC model [31], which allows for divergent selection pressures at one site class between different clades of a phylogeny or between paralogs. A prediction of this model is that one gene, in a gene family, can experience reduced functional constraint, via genetic redundancy, allowing the accumulation of mutations that will alter protein function, particularly at a time when the environment changes. First, the M0 model was used to obtain the global constrained estimate of ω of 0.096 for the entire UCP phylogeny (Figure 2). We then ran the M2 branch model using each UCP Eutherian lineage as the foreground branch. The ω estimate for the UCP1 Eutherian branch was 0.680, and was a significantly better fit to the data than the M0 model (p-value < 0.001), as was found previously when only the UCP1 phylogeny was analyzed (Figure 1, Relaxed Constraint on the Eutherian Lineage). For the UCP2 locus, the ω estimate for the Eutherian lineage was 0.044, which is significantly smaller than the global ω estimate (p-value = 0.03). The ω estimate for the UCP3 Eutherian lineage was 0.081, which is not significantly different from the global ω estimate (p = 0.68). Thus, UCP1 (but not UCP2 or UCP3) exhibits a significantly higher ω value on the Eutherian lineage in the context of the entire UCP phylogeny, consistent with our previous observations and relaxed functional constraint.

We then fit our data to the MC model with five different clades, A-E, set as the foreground branches (Figure 2). The MC model (clade model C) has five parameters (p_0_, p_1_, ω_0_, ω_2 _and ω_3_) estimated. There are three site classes; site class 0 are defined sites of purifying selection where ω_0 _is estimated to be between 0 and 1 (p_0 _= proportion of class 0 sites), site class 1 are neutral sites with ω_1 _set equal to 1 (p_1 _= proportion of class 1 sites), and site class 2 is the divergent site class where there is an independent estimate of ω for the background (ω_2_) and foreground (ω_3_) branches (proportion of sites in class 2: p_2 _= 1 - p_0 _- p_1_). The LR test for each of these analyses is the comparison of the MC model to the M1a(neutral) sites model, in which there are two site classes across the entire UCP phylogeny (site class 0 is under purifying selection and ω is estimated from the data; site class 1 is neutral and ω is set equal to 1). The M1a(neutral) model estimates that 90% of the sites are under strong purifying selection with a ω estimate of 0.08. Each MC analysis provided a significantly better fit to the data than the M1a(neutral) model (Table 2). The parameter estimates (Table 2) are very similar across analyses, suggesting that the model is indeed robust to altering foreground and background branches. To summarize, overall about 60% of the sites in the UCP phylogeny are under strong purifying selection, about 1% of the sites are evolving neutrally, and about 39% of the sites are under divergent selection pressures between UCP1 and its paralogs. The results for clades B, C, and D (Table 2, foreground ω) suggest that there has been more purifying selection across the UCP2 phylogeny than that of the UCP3.

**Table 2:** MC clade model parameter estimates, LRT, and p-value

**Clade**	**p_0_**	**ω_0_**	**p_2_**	**Background ω**	**Foreground ω**	**2Δℓ**	**p**
**A_UCP1**	0.57	0.02	0.39	0.17	0.24	1095	<0.001
**B_UCP2/3**	0.58	0.02	0.39	0.22	0.18	1087	<0.001
**C_UCP2**	0.58	0.02	0.38	0.22	0.14	1099	<0.001
**D_UCP3**	0.59	0.02	0.37	0.19	0.22	1084	<0.001
**E_Eutherian_UCP1**	0.56	0.02	0.4	0.17	0.29	1119	<0.001

p_0 _is the estimated proportion of sites in site class 0 (purifying selection), ω_0 _is the estimated ω value for site class 0; p_2 _is the estimated proportion of sites in site class 2 (divergent selection), Background ω (ω_2_) is the estimated ω value for divergent sites on the background branches; Foreground ω (ω_3_) is the estimated ω value for divergent sites on the foreground branches; 2Δℓ is the LR test statistic for comparing the MC and M1a(neutral) models; p is the p-value of the LR test.

The model with the highest log likelihood is with the Eutherian UCP1 clade (clade E) set as the foreground branch. Similar results are obtained when the entire UCP1 clade (clade A) is set as the foreground branch. However, the log likelihood is lower and the ω_3 _estimate for the foreground divergent class is larger when only considering the Eutherian UCP1 clade (clade E). The ω_3 _observed (0.24) for the entire UCP1 clade (clade A) may largely reflect the divergent selection pressures in the Eutherian clade (ω_3 _= 0.29), with the marsupial and fish lineages reducing the ω_3 _estimate for the foreground branch in the entire UCP1 phylogeny (clade A). Furthermore, it is the Eutherian UCP1 lineage itself that is driving the functional divergence of the Eutherian UCP1 clade. Repeating the MC model but setting the Eutherian UCP1 lineage (E) (i.e. just branch E, not the clade) as the foreground branch, an ω of 0.87 is estimated for divergent sites, while background lineages have an ω of 0.18, where p_2 _= 0.390 (2Δℓ = 1119, p < 0.001; identical ℓ to Clade E (Table 2)). In conclusion, these analyses indicate that the UCP1 phylogeny exhibits large divergent selection pressures, which may principally involve the Eutherian UCP1 lineage, consistent with the possible acquisition of the new function of UCP1 through neutrally evolving, relaxed constraints.

**Figure 3:**
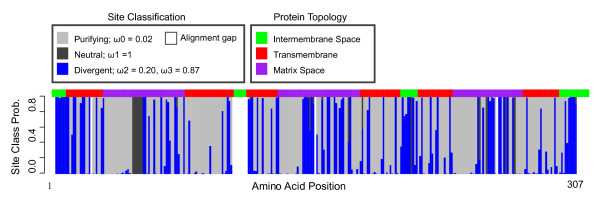
**A bar plot of site class probabilities for each codon, represented by amino acids in the figure**. Site classifications and their omega estimate are defined in the figure. The protein topology, defined as intermembrane, transmembrane and matrix space protein portions was placed above the barplot. Topology was taken from [56].

The BEB method is implemented in each analysis to determine the probability [26] of each site belonging to site class 0 (purifying), site class 1 (neutral), or site class 2 (divergent). Site classifications are consistent across each analysis. Figure 3 is a plot of site class probability, i.e. the probability of each codon belonging to one of the three site categories, namely purifying, neutral or divergent. Approximately 40% of all sites are under divergent selection pressure, with nonsynonymous substitutions accumulating at a rate ~1.7× greater in the UCP1 Eutherian clade (clade E) relative to its paralogs, as compared to synonymous substitutions. Sites categorizations are nearly identical when the MC model is run with either the Eutherian UCP1 clade or the Eutherian UCP1 lineage set as the foreground branch (5% discrepancy in site classification between analyses). It is only the ω estimations that are largely different for the divergent class between the two analyses (0.29 vs. 0.87). In contrast to the Eutherian clade analysis, the Eutherian lineage analysis has a nonsynonymous substitution rate that is ~5× greater than that of all other lineages in the entire phylogeny. Divergent sites are found across the entire protein and in all functional domains, assigned by their topology to regions located in the intermembrane, transmembrane, and matrix space. Notably, the protein regions exposed to the intermembrane space are significantly over-represented with divergent sites (χ^2 ^= 26.32, p-value = 0.0005, p computed by Monte Carlo simulation). This result suggests that functional constraints are more relaxed for the regions of the protein exposed to the intermembrane space in Eutherians. This may have enabled the recruitment of novel interactions with proteins which potentially regulate the activity of UCP1.

### Positive selection following duplication

In addition to any selection on UCP1 for NST in Eutherians, there may have been positive selection on other sites in any of the UCP loci following the gene duplications that created them. We therefore used the UCP phylogeny (Figure 2) and the branch-sites model (MA) to test lineages A (UCP1), B (UCP2/3), C (UCP2), and D (UCP3) for positive selection acting on only a few sites. Again, LR tests were used to compare the MA model to the MA1 model, in which the ω estimate for site class 2 is set to 1, to assess if the MA model with positive selection is significantly better than the MA1 model of neutrality. No robust statistical evidence for selection was discovered for any basal UCP lineage. Statistical evidence for selection was identified for UCP1 (A), UCP2/3 (B) and UCP2 (C) when the analysis was carried out using the MUSCLE alignment (Table 3), but these results were not supported by the T-Coffee alignments, and hence any conclusions about positive selection on any of the UCP loci following gene duplication depend on the alignment.

**Table 3:** MA branch-sites parameter estimates following gene duplication

**Lineage**	**p0**	**ω0**	**p2**	**ω2**	**Selected Sites**	**2Δℓ**	**p**
**A_UCP1**	0.887	0.078	0.022	468.880	>90% 256M	6.803	0.009
**B_UCP2/3**	0.875	0.076	0.034	999.000	>80% 148A, 274M, >90% 120L	7.709	0.005
**C_UCP2**	0.886	0.076	0.021	14.950	>95% 207A, 300A	4.780	0.028
**D_UCP3**	0.878	0.076	0.029	2.870	>70% 130C	0.028	0.868

p_0 _and p_2 _are the estimated proportion of sites, and ω_0 _and ω_2 _are the estimated ω values for site class 0 (purifying selection) and site class 2 (positive selection), respectively; Selected sites are those with a large posterior probability of belonging to the selected site category.

### Codon usage bias

A recently developed codon substitution model, FMutSel, explicitly models mutational biases and selection at silent codon sites [32]. This model was compared to the null model, FMutSel0, which only models mutational biases, and does not include the additional codon fitness parameter in FmutSel which models selection on silent sites. The LRT between these two models has 41 degrees of freedom, resulting from the parameter rich FMutSel model (60 parameters). It has been suggested that codon usage bias could have a drastic effect on estimation of dN and dS [22,33]. The species tree for each UCP locus was used in these analyses, not the full UCP phylogeny found in Figure 2. We find that each of the UCP loci has statistically significant evidence for selection at silent sites (Table 4). This result is not surprising, as about 90% of 5,369 genes tested reject the null model, indicating that selection on silent sites is a common phenomenon across the mammalian genome [32]. Thus, codon usage bias is widespread and silent sites are not neutrally evolving. This conclusion, however, does not discredit the use of ω (the comparison of dN and dS) as a means of identifying sites of selection. Selection on the protein alters the rate of evolution at replacement sites, and it is this rate of change before and after selection that is contrasted. Thus, it does not matter if the rate of substitution at silent sites is driven by mutation or selection [32].

**Table 4:** FMutSel LRT and parameter estimates with the M0 model

**Locus**	**FMutSel ℓ**	**FMutSel0 ℓ**	**2Δℓ**	**p-value**	**ω**	**P+**	**S+**	**S-**
UCP1	-10029.62	-10118.34	177.44	<0.001	0.129	0.40	1.42	-1.58
UCP2	-8657.37	-8777.49	240.24	<0.001	0.074	0.31	1.90	-2.44
UCP3	-8857.95	-9004.26	292.62	<0.001	0.099	0.41	1.57	-1.93

FMutSelℓ is the log likelihood of the FMutSel substitution model; FMutSel0ℓ is the log likelihood of the FMutSel0 substitution model; 2Δℓ is the LR test statistic for comparing these two models; p is the p-value of the LR test; ω is the estimated ω value for the designated gene phylogeny using the global M0 model; P+ is the proportion of advantageous mutations that could occur across the gene; S+ is the selection coefficient for advantageous mutations; S- is the selection coefficient for disadvantageous mutations.

Interestingly, even though the FmutSel model was a much better fit to the data than the null model for all three loci, the statistical significance of the difference between the models for the UCP2 and UCP3 loci are orders of magnitude larger than for the UCP1 locus (Table 4). This may suggest that selection on silent sites has been reduced across the UCP1 phylogeny. The proportion of advantageous mutations (P+) estimated in the UCP phylogenies is about 0.3 – 0.4, similar to the average estimate of 0.39 across 5,639 genes [32]. In contrast, the estimated average selection coefficient for advantageous (S+) and disadvantageous (S-) mutations (Table 4) for each of these UCP loci are greater than three times the average selection coefficient for other genes [32]. Thus, mutations at silent sites in these three loci would seem to be either highly preferential or highly deleterious, as compared to the genome average.

## Discussion

It has long been thought that UCP1 had evolved in Eutherian mammals in conjunction with the evolution of brown adipose tissue. Recent discoveries of UCP1 orthologs in fish and non-Eutherian mammals have now discredited this idea [1,2,17], as fish and most non-Eutherian mammals do not contain brown adipose tissue where UCP1 plays a major role in thermogenesis. Furthermore, the recent discovery of UCP1 localized in adipose tissue in a marsupial species, where it is also regulated in response to cold acclimatization [1] has forced a reevaluation of the evolution of non-shivering thermogenesis. In particular, when did the biochemical properties of UCP1 which confer the capability of thermogenesis evolve?

In this study we have used codon substitution models implemented through a maximum likelihood framework to estimate the rate of evolution at silent and replacement sites in UCP1 and its paralogs, UCP2 and UCP3. Different models were used to investigate variation in the rate of evolution between lineages of a phylogeny, between clades of a phylogeny, and to estimate ω for specific lineages and sites across phylogenies. Our objective was to determine the mode of evolution on the UCP1 Eutherian lineage, to illuminate possible evolutionary consequences of novel functions necessary for NST. We observed a significant, five-fold increase in the rate of UCP1 evolution on the Eutherian lineage, but found no strong statistical evidence for directional selection. Yet, it should be noted that the power of these analyses are dependent on the magnitude of the selection pressure and the proportion of sites affected [34,35]. These models do have underlying assumptions, namely no variation in branch and site ω values. As we have demonstrated, variation among branch and sites is observed in the UCP1 phylogeny. While large deviations from these assumptions will affect the power of the analysis, the MA model does seem to be robust to moderate deviation in these assumptions [34]. As such, relaxed functional constraint is most consistent with the molecular evolutionary analyses of the UCP data. Further, our observation of strong purifying selection being the primary mode of evolution throughout the UCP phylogeny is consistent with the findings of a recent study published while this paper was under review [36].

Our conclusion is in contrast to a previous study which implemented a heuristic branch model and concluded that the UCP1 Eutherian lineage had experienced positive selection on replacement sites [18]. That study estimated 82.8 nonsynonymous and 4.3 synonymous substitutions on the Eutherian branch using a least-squares approach, whereas we estimate 88.5 nonsynonymous and 47.5 synonymous substitutions respectively. We repeated the analyses in [18], using their methodology, and obtained essentially the same results they did (data not shown). To determine why these two methods are giving drastically different results, we simulated the evolutionary history of UCP1 using the branch-lengths given by the M0 global model, the global ω estimate of 0.1134, and the Evolver program in the PAML 4a package (Additional file 2). We then estimated ω for the Eutherian lineage using both the heuristic method [18] and the ML (M2) model used in this study. The resulting ω estimate for the ML model was 0.1135, virtually identical to the value used in the simulations, while the ω estimate for the heuristic approach was 1.239. We repeated the simulation, setting ω = 0.5 for the Eutherian lineage and ω = 0.1 for all other lineages, which mirrors the observed data. The resulting ω estimate for the Eutherian lineage was 0.47 based on the ML model and 7.75 based on the heuristic approach. In each simulation the heuristic approach greatly under-estimated the number of synonymous substitutions. We conclude that the contradiction between our results and the previous study [18] results from the poor performance of the heuristic least-squares method on trees and lineages with long branch lengths. It appears that least-squares method drastically underestimate the number of synonymous substitutions [37].

An alternative to relaxed functional constraint as an explanation for the increased rate of evolution on the UCP1 Eutherian lineage would be a decrease in the effective population size (N_e_) of the ancestor of all Eutherian mammals. However, a change in N_e _along the Eutherian lineage would affect all loci in the genome. As such the UCP1 paralogs, UCP2 and UCP3, should also reflect any possible changes in N_e_. Yet these UCP1 paralogs do not exhibit a rate increase, so a decrease in N_e _can be excluded. Therefore it remains that the best explanation for the increase in UCP1 evolution along the Eutherian lineage is relaxation of functional constraints, possibly allowing functional divergence through neutral processes.

Assuming that UCP1 did indeed experience relaxation of functional constraints on the Eutherian lineage, what may have caused this? There are several possible explanations, which are not mutually exclusive. First, it has been documented that the physiological relevance of thermogenic function varies across Eutherians, with NST being most efficient in small mammals and hibernators, particularly those which inhabit environments with long cold seasons [38,39]. If the ancestor of all Eutherians was a large species where NST was not important, or a species which inhabited a moderate environment, functional constraints on UCP1 could have been relaxed. The ancestor of all extant Eutherians lived ~150–100 MYA, during the late Jurassic and early Cretaceous [40]. In general mammals of this period are thought to have been small in size [41] and this era was also generally warmer than today [42,43]. Thus it is possible that constraints on UCP1 were reduced at some time point prior to the Eutherian radiation, some 100 MYA. Second, it could be that some function(s) of UCP1 in Eutherians was taken over by UCP1's paralogs, which would also reduce the level of constraint. It is certain that UCP1 in Eutherians must have had a role in some other tissue(s) prior to becoming an obligate BAT protein. Genes expressed in many tissues tend to be more constrained than those expressed in one or a few tissues [44]. Thus, the release of UCP1 from its role in other tissues would allow UCP1 to evolve tissue specificity to BAT, decrease the selective constraint on UCP1, and allow for genetic co-option (the altering of a genetic system for a new use). For example, as shown in Table 1, the ω estimate (ω_0_) assigned to site class 1 (strong purifying selection) suggests that there has been more selective constraint on UCP2, which is expressed in many tissues, than on UCP3, which is expressed in just a few tissues [15,16]. The function(s) of UCP2 and UCP3 are currently uncertain; determining their function(s) would certainly aid in understanding UCP1 evolution.

In the process of genetic co-option or functional gene divergence it is not necessary for directional selection to act on protein evolution in order for divergent/novel functions to evolve. Constraint on a protein can be relaxed when a paralog is available to fulfill the function of that protein. As such, evolution can then act to use existing gene functions for a new purpose. The prediction of heterogeneity in selection pressures between gene paralogs exemplifies the divergent selection pressures that allow the evolution of divergent functions. We examined the potential of genetic co-option, i.e. functional divergence of UCP1, through the implementation of the MC clade model [31]. We found that about 40% of the sites were under divergent selection pressures, with the UCP1 clade experiencing a greater number of substitutions at replacement sites relative to silent sites (Figure 3). Moreover, intermembrane sites are over-represented in the divergent site category, which may suggest that intermembrane interactions have diverged in Eutherian UCP1. Regardless, it is apparent that there has been tissue-specific functional divergence of UCP1 in BAT.

Cold-induced UCP1 expression was recently detected in adipose tissue of the interscapular region of the Australian fat-tailed dunnart (*Sminthopsis crassicaudata*), a dasyurid marsupial species [1]. Further, the adipose tissue in which it was found was described as being an archetypal brown adipose tissue. As such, these findings challenge the view of NST as an exclusive property of Eutherians. However, this finding is unique to this particular marsupial, as cold-induced UCP1 expression was not found in the dasyurid marsupial *Antechius flavipes *(yellow-footed Antechinus) or the South American didelphid marsupial *Monodelphis domestica *(gray short-tailed opossum). Furthermore, there is currently no evidence for a thermogenic role of UCP1 in the fat-tailed dunnart. Its regulation and expression is suggestive of a thermogenic role, yet no evidence of non-shivering thermogenesis or "adaptive-NST", i.e. an adaptive organismal response to the cold, has been demonstrated. As such, if NST is determined to be present in the fat-tailed dunnart, it would most likely be a case of parallel evolution or multiple lineage loss of NST, as deduced from phylogenetic inference and given that these findings are restricted to a single non-Eutherian species.

Nevertheless, since UCP1 in the fat-tailed dunnart does show a response to cold and norepinephrine [29] similar to that found in Eutherians, it may be that any truly novel function of UCP1 started evolving in the Therian (marsupial and Eutherian) lineage. We therefore tested for selection on UCP1 on this lineage and found strong evidence for positive selection, and we identified two sites (79P and 178T) which may be responsible for any novel UCP1 function. These two sites should be the target of further functional investigations. Moreover, it is not clear if UCP1 in marsupials is truly functionally identical to UCP1 in Eutherians; more studies are needed to clarify this issue.

## Conclusion

In agreement with previous studies, we find evidence for acceleration in the evolution of UCP1 on the Eutherian lineage. However, in contrast to previous studies, our analyses indicate that relaxed constraints on UCP1, rather than directional selection, seem to be responsible. We suggest that this relaxation of constraints on UCP1 may have allowed the acquisition or enhancement of the (potentially) novel role of UCP1 in NST in Eutherians, as supported by the divergent selection pressures identified in the UCP phylogeny. Currently, the most likely hypothesis for the origin of NST hinges upon the evolution of BAT with high mitochondrial oxidative capacities. For this requirement to be meet evolution needs a tissue with many mitochondria which in turn have a high expression level of UCP1 and complexes I-IV of the electron transport chain. This combination can then induce a high mitochondrial oxidative capacity which will lead to heat production (i.e. NST). As such, we hypothesize that UCP1 in Eutherians and non-Eutherians share similar or identical biochemical properties. To test this hypothesis the biochemical properties of UCP1 in non-Eutherians need to be elucidated.

## Methods

### Coding sequence sources

Coding sequences (cds) for UCP1, UCP2 and UCP3 were obtained from the NCBI CoreNucleotide and Ensembl  databases. A table with species names, abbreviations and accession numbers can be found in supplementary materials (Additional file 1).

### Data analysis

Coding sequences were imported into MEGA 3.1 [45] and converted into amino acid sequences, which were then aligned with MUSCLE 3.6 [46] and T-Coffee 3.8 [47] and exported in CLUSTAL W format. The amino acid alignment was subsequently transformed into an aligned cds fasta file with a custom PERL script. The accepted species tree [40,48] was generated by hand and gene trees were generated using various nucleotide and amino acid substitution models in PHYLIP [49]. The gene trees analyzed included: neighbor joining (NJ) trees from nucleotide sequence distances (dnadist; F84, Kimura and Jukes-Cantor substitution models) with a gamma distributed rate variation and a transition/transversion bias (κ) of 2.0; a DNA maximum likelihood (dnaml) tree with κ = 2.0, rate variation among sites, global rearrangements, and randomized input; and lastly a NJ tree from protein distances (protdist) was generated using the JTT and PAM substitution models and a gamma distributed rate variation. Maximum likelihood analyses were performed using the aligned cds and the CODEML program found in the PAML 4a package [19]. The species tree and generated gene trees were analyzed with the CODEML program of the PAML 4a package, which implements the re-sampling estimated log-likelihood (RELL) method, and the likelihood ratio tests K-H and S-H. These are nonparametric likelihood ratio tests, which test if two phylogenetic trees differ significantly in how well they fit the data. As it is not appropriate to use a maximum likelihood tree with the K-H test [50] we focused on the results from the S-H test and the RELL method. Finally, CODEML of the PAML 4a package was also used to estimate the ratio of non-synonymous or replacement changes per site/synonymous changes per site, also known as the dN/dS ratio or the omega value (ω). CODEML implements a maximum likelihood (ML) method of estimating ω [51], using a variety of codon substitutions models and estimation of κ, and accounts for base frequency biases at codon positions. Different site [52,53], branch [54,55], branch-sites [22,23,26], and clade models [31] were implemented in estimating the ω value. In this study we used the F3X4 and F61 models of codon frequencies [51], as well as the recently developed FMutSel model [32], which models mutational biases and introduces a parameter of codon fitness to model selection at silent sites. The F3X4 and F61 codon models differ in the manner in which they estimate each codon's equilibrium frequency. The F3X4 model derives equilibrium codon frequencies from the frequencies of the three nucleotides at the three codon positions, while the F61 model uses each codon as a free parameter and constrains the sum to one. Analyses such as these allow the determination of the type of evolution (neutrality, negative selection, or positive selection) which predominates across the entire phylogenetic tree, across each site in the sequence, on particular branches, at particular sites on particular branches, or at particular sites across clades. In these analyses an ω value equal to one is indicative of neutral evolution, ω greater than one is indicative of positive selection, and ω less than one is indicative of negative or purifying selection.

## Authors' contributions

MK and MJ initiated the study; DH and MS conceived the study design; DH performed the analyses and analyzed the data; DH, MS and MK wrote the manuscript. All authors approved the final manuscript.

## Supplementary Material

Additional file 1 **Accession Numbers**. Table of accession numbers including: list of species, species abbreviations, and accession numbers for sequences used in the study. Click here for file

Additional file 2 **UCP1 Simulations**. Description and results of UCP1 simulations and ω estimations using heuristic and maximum likelihood approaches. Click here for file
