# Supplementary material for: Molecular evolution of UCP1 and the evolutionary history of mammalian non-shivering thermogenesis
Source: BMC Evol Biol. 2009 Jan 7;9:4. doi: 10.1186/1471-2148-9-4 (PMC2627829; doi:10.1186/1471-2148-9-4)
Supplement: Additional file 1 — Accession Numbers. Table of accession numbers including: list of species, species abbreviations, and accession numbers for sequences used in the study. [file 1471-2148-9-4-S1.pdf]

Species	Abbreviation	Common Name	UCP1	UCP2	UCP3
Antechnius flavipes	Afl	yellow-footed Antechnius		AY233003	AY519198.1
Bos torus	Bta	cow	XM_616977	XM_614452	NM_174210
Canis familiaris	Cfa	dog	NM_001003046	NM_001003048	NM_001003047
Ctenopharyngodon idella	Cid			AY948546	
Cyprinus carpio	Cca	common carp	AY461434	AJ243486	
Danio rerio	Dre	zebrafish	NM_199523	ENSDART00000063359	Klingenspor Lab
Dicrostonyx groenlandicus	Dgr	lemming	AF515781	AY484518	
Echinops telfairi	Ete	tenrec	ENSETET00000010923		
Equus caballus	Eca	horse		NW_001799731	
Erinacaeus europaeus	Eeu	hedgehog	ENSEEUG00000005182	ENSEEUG00000007994	
Eupetomena macroura	Ema	hummingbird			AF255729
Felix catus	Fca	cat		ENSFCAG00000005031	ENSFCAG00000005033
Gasterosteus aculeatus	Gac	stickleback		ENSGACT00000026955	BT027074
Gallus gallus	Gga	Chicken			ENSGALT00000027985
Homo sapiens	Hsa	human	NM_021833	NM_003355	NM_003356
Leuciscus cephalus	Lce	European chub		AY368268	
Macaca mulatta	Mamul	Rhesus macaque	XM_001090457	ENSMMUG00000002605	ENSMMUG00000010317
Mesocricetus auratus	Mau	golden hamster	X73138		
Microcebus murinus	Mmur	gray mouse lemer		ENSMICT00000012697	ENSMICT00000012706
Monodelphis domestica	Mdo	opossum	ENSMODT00000000201	ENSMODT00000009488	ENSMODT00000009440
Mus musculus	Mmu	mouse	NM_009463	NM_011671	AF032902
Myotis lucifugus	Mlu	brown bat	ENSMLUT00000009572	ENSMLUT00000012109	ENSMLUT00000012118

Ochotona princeps	Opr	pika		ENSOPRT00000010420	
Ochotona dauurica	Oda	Daurian Pika	AB283043		
Oncorhynchus mykiss	Omy			DQ295326 / DQ295328	
Ornthorhynchus anatinus	Oan	platypus	ENSOANT00000024080	ENSOANT00000011476	ENSOANT00000012331
Oryctolagus cuniculus	Ocu	rabbit	X14696		
Otolemur garnettii	Oga	bushbabby	ENSOGAT00000006809	ENSOGAT00000001699	ENSOGAT00000002909
Pan troglodytes	Ptr	chimpanzee	ENSPTRT00000030601	ENSPTRT00000007559	ENSPTRT00000007562
Phodopus sungorus	Psu	Djungarian hamster	AF271263	AF271264	AF271265
Rattus norvegicus	Rno	rat	NM_012682	NM_019354	NM_013167
Sminthopsis crassicaudata	Scr	Fat Tailed Dunnart	EF622232		
Suncus murinus	Smu	Asian house shrew	AB244816		
Sus scrofa	Ssc	pig		NM_214289	NM_214049
Tetraodon nigroviridis	Tni	pufferfish	CR640550	Klingenspor Lab	Klingenspor Lab
Takifugu *rubripes*	Tru	pufferfish	SINFRUT00000155540		Klingenspor Lab
Tupaia belangeri	Tbe	tree shrew	ENSTBET00000000042	ENSTBET00000001469	
Zoarces viviparus	Zvi	viviparous blenny		AY625191	
Xenopus tropicalis	Xtr	western clawed frog	ENSXETT00000032640	ENSXETT00000055772	Klingenspor Lab
