# Supplementary material for: Molecular evolution of UCP1 and the evolutionary history of mammalian non-shivering thermogenesis
Source: BMC Evol Biol. 2009 Jan 7;9:4. doi: 10.1186/1471-2148-9-4 (PMC2627829; doi:10.1186/1471-2148-9-4)
Supplement: Additional file 2 — UCP1 Simulations. Description and results of UCP1 simulations and ω estimations using heuristic and maximum likelihood approaches. [file 1471-2148-9-4-S2.pdf]

Supplementary Text 1:

Evolver Simulations

The primary conclusion presented in this study contradicts that of a previous study [1]: we conclude that the long Eutherian branch in the UCP1 phylogeny is the result of relaxed functional constraints, while the previous study concluded that directional selection on UCP1 in the ancestor of Eutherians was responsible. The major methodological difference in the two studies is the approach used in estimating evolutionary rates. The previous study employed a heuristic approach and a least-squares distance estimator, while we used a maximum likelihood approach. These two methods give similar estimates for the number of nonsynonymous changes (82.8 and 88.5, respectively) along the Eutherian branch, but very different estimates of the number of synonymous changes (4.3 and 48.5, respectively). To determine if one approach is sensitive to large evolutionary distances and thus long branch lengths between sequences, we simulated the evolutionary history of UCP1 using the "Evolver" program in the PAML 4a package. Evolver can simulate coding sequence data using a user-specified tree with branch lengths. Four parameters are passed to the program: (1) the tree and branch lengths, which were derived from the M0 model used in this study; (2) the ω value, 0.1135, which is held constant for every lineage and every site in the tree; (3) the codon frequency table which is derived from observed frequencies; and (4) the transition/transversion ratio (κ), , which was set to the estimated value of 2.13.

Simulated sequence data and the predefined user-tree were then used to estimate ω for the Eutherian lineage, using both the heuristic approach [1] and the maximum likelihood approach used in this study. Specifically, the Bn-Bs program [2] was used to

estimate non-synonymous and synonymous branch lengths in a heuristic manner as was done previously [1]. This method resulted in ω=1.239, where bn = 0.05824 and bs =0.04699. Fitting the data to the maximum likelihood (M2) model estimated ω=0.1135 for the Eutherian lineage. These results demonstrate that the heuristic approach is overestimating the ω value for the Eutherian lineage.

A second simulation was performed to create data which more closely reflect the observed data for UCP1. In the second simulation, two ω values were passed to the Evolver program. All lineages but the Eutherian lineage were given an ω of 0.1, while the Eutherian lineage was given a unique ω of 0.5. The same tree, branch lengths, codon frequency table, and κ value were used in this simulation; only the ω value for the Eutherian lineage was changed. Simulated data were again passed to the Bn-Bs program and to the M2 model of the ML approach. The heuristic approach estimated an ω of 7.75 for the Eutherian branch, which is greatly inflated compared to the ω value used in the simulations (0.5), but is similar to that estimated previously (6.4) from the UCP1 data [1]. By contrast, the M2 model estimated an ω of 0.4692, close to the value used in the simulations. It therefore appears that the heuristic approach used previously is inappropriate for the UCP1 data, as the method drastically underestimates dS, thereby falsely inflating the dN/dS ratio and creating a type I error.

References

1. Saito S, Saito CT, Shingai R: **Adaptive evolution of the uncoupling protein 1 gene contributed to the acquisition of novel nonshivering thermogenesis in ancestral eutherian mammals**. *Gene* 2008, **408**(1-2):37-44.

2. Zhang J, Rosenberg HF, Nei M: **Positive Darwinian selection after gene duplication in primate ribonuclease genes**. *Proc Natl Acad Sci U S A* 1998, **95**(7):3708-3713.
